# Meta-analyses of FibroTest diagnostic value in chronic liver disease

**DOI:** 10.1186/1471-230X-7-40

**Published:** 2007-10-15

**Authors:** Thierry Poynard, Rachel Morra, Philippe Halfon, Laurent Castera, Vlad Ratziu, Françoise Imbert-Bismut, Sylvie Naveau, Dominique Thabut, Didier Lebrec, Fabien Zoulim, Marc Bourliere, Patrice Cacoub, Djamila Messous, Mona Munteanu, Victor de Ledinghen

**Affiliations:** 1 Service d'Hépato-Gastroentérologie, Groupe Hospitalier Pitié-Salpêtrière, Université Paris VI, CNRS ESA 8067 Paris, France; 2 Laboratoire Alphabio, Marseille, France; 3 Service d'Hepato-Gastroenterologie, Hopital Haut Leveque, Pessac, France; 4 Laboratoire de Biochimie, Groupe Hospitalier Pitié-Salpêtrière, Paris, France; 5 Service d'Hépato-Gastroentérologie, Hopital Antoine Beclere, Clamart, France; 6 INSERM, U773, Centre de Recherche Biomedicale Bichat-Beaujon CRB3, Service d'Hépatologie, Hopital Beaujon, Paris, France; 7 INSERM U271, Virology laboratory, University Hospital Center, Lyon, France; 8 Service d'Hepato-Gastroenterologie, Hopital Saint-Joseph, Marseille, France; 9 Service de Medecine Interne, Groupe Hospitalier Pitié-Salpêtrière, Université Paris VI, Paris, France; 10 Biopredictive, Paris, France

## Abstract

**Background:**

FibroTest (FT) is a biomarker of liver fibrosis initially validated in patients with chronic hepatitis C (CHC).

The aim was to test two hypotheses, one, that the FT diagnostic value was similar in the three other frequent fibrotic diseases: chronic hepatitis B (CHB), alcoholic liver disease (ALD) and non-alcoholic fatty liver disease (NAFLD); and the other, that the FT diagnostic value was similar for intermediate and extreme fibrosis stages.

**Methods:**

The main end points were the FT area under the ROC curves (AUROCs) for the diagnosis of bridging fibrosis (F2F3F4 vs. F0F1), standardized for the spectrum of fibrosis stages, and the comparison of FT AUROCs between adjacent stages. Two meta-analyses were performed: one combining all the published studies (random model), and one of an integrated data base combining individual data. Sensitivity analysis integrated the independency of authors, lenght of biopsy, prospective design, respect of procedures, comorbidities, and duration between biopsy and serum sampling.

**Results:**

A total of 30 studies were included which pooled 6,378 subjects with both FT and biopsy (3,501 HCV, 1,457 HBV, 267 NAFLD, 429 ALD, and 724 mixed). Individual data were analyzed in 3,282 patients. The mean standardized AUROC was 0.84 (95% CI, 0.83–0.86), without differences between causes of liver disease: HCV 0.85 (0.82–0.87), HBV 0.80 (0.77–0.84), NAFLD 0.84 (0.76–0.92), ALD 0.86 (0.80–0.92), mixed 0.85 (0.80–0.93). The AUROC for the diagnosis of the intermediate adjacent stages F2 vs. F1 (0.66; 0.63–0.68, n = 2,055) did not differ from that of the extreme stages F3 vs. F4 (0.69; 0.65–0.72, n = 817) or F1 vs. F0 (0.62; 0.59–0.65, n = 1788).

**Conclusion:**

FibroTest is an effective alternative to biopsy in patients with chronic hepatitis C and B, ALD and NAFLD. The FT diagnostic value is similar for the diagnosis of intermediate and extreme fibrosis stages.

## Background

Fibrotest (FT) is a biomarker of liver fibrosis which was initially validated in patients with chronic hepatitis C (HCV) [1] and then in the three other common fibrotic liver diseases: [2] chronic hepatitis B (HBV) [3,4], alcoholic liver disease (ALD) [5-7] and non-alcoholic fatty liver disease (NAFLD) [8].

FT is widely used as a non invasive alternative to liver biopsy, with 190,000 tests ordered between September 2002 and April 2007 (Biopredictive data on file, Jean Marie Castille, personal communication); however, two main critiques are often made by experts: 1) FT has been mainly studied in chronic hepatitis C, and 2) the FT diagnostic value is lower for intermediate fibrosis stages (bridging vs. non bridging fibrosis) than for extreme stages (no fibrosis or cirrhosis)[9,10]. In this latter critique, which is also true for liver biopsy, there is a risk of confusion between adjacent stages and intermediate stages or an absence of taking into account the prevalence of fibrosis stages defining advanced and non-advanced fibrosis [11,12].

The aim of this meta-analysis was to test two hypotheses, first, that the FT diagnostic value was similar in patients with HCV and in patients with the three other frequent fibrotic diseases; and second, that the FT diagnostic value was similar for intermediate and extreme stages.

## Methods

### Design

Two meta-analyses were performed; one combined all the published studies (random model), and the other used an integrated database combining individual data provided by authors.

To select published studies we used the Standards for Reporting of Diagnostic Accuracy (STARD) criteria and the Cochrane Database of Systematic Reviews (CDSR) methods [13]. Key STARD criteria include factors such as whether: 1) the study population was relevant to the clinical question being addressed; 2) there was a careful description of the population from which the patients were drawn, as well as actual inclusions and exclusions; 3) recruitment and the mode of sampling were carefully described; 4) researchers interpreting the non-invasive test were blinded to the reference test result; and 5) sufficient data were provided to complete a 2 × 2 table of true and false positive and negative diagnoses. Studies published only with an abstract provided insufficient data and were excluded  [14].

### Search strategy

We searched MEDLINE with the key word "FibroTest". We hand-searched key journals (*Gastroenterology, Hepatology, Journal of Hepatology, Gut, Journal of Viral hepatitis *and *American Journal of Gastroenterology*) from February 2001 to April 2007 to validate the search, as well as the abstract books of the American Association and European Association for the Study of Liver Disease annual meetings.

### Inclusion and exclusion criteria

Two reviewers (a hepatologist and a hepatologist-statistician) independently assessed the papers with predetermined STARD criteria. Disagreements were resolved through discussion with a third reviewer. The decision as to inclusion or exclusion was not related to results.

We excluded all studies except those that: included patients with chronic liver diseases; stated that all patients had had the FT and liver biopsy; provided data for true positives and negatives, false positives and negatives and AUROCs for advanced fibrosis; stated that the FT had been assessed blind to the biopsy; and stated the method used for defining the degree of fibrosis. We were careful to avoid including data from duplicate publications.

### Data extraction

To allow comparisons between causes of liver disease in the studies, we categorized them into 5 classes: patients with CHC, CHB, ALD, NAFLD and mixed causes.

We extracted the following, when possible, from the published studies and from the integrated database for the sensitivity analyses: study design (prospective or retrospective); analytical procedures (fresh serum and compliance with the recommended procedures or not); spectrum of disease (patients with elevated or normal transaminases); co-morbidity (several morbidities including HCV, HBV, alcohol consumption, HIV coinfection, presence/absence of renal disease); and whether the study was performed by the FT inventor group (yes, no, mixed groups including inventor). Patient inclusion was never dependent on the result of the non-invasive test under investigation.

### Statistical analysis

#### Comparison of FT diagnostic values between different chronic liver diseases

The main end point was the FT value for the diagnosis of advanced fibrosis (bridging fibrosis or stages F2, F3, F4 according to the METAVIR scoring system [15-17]), as assessed by the area under the receiver operating characteristics curve (AUROC)].

#### Comparison of FT diagnostic values between adjacent stages

The main endpoint was the comparison of the FT AUROCs between adjacent stages: either between two adjacent intermediate stages F2/F1 or between two adjacent extreme stages F4/F3 and vs. F1/F0.

#### Statistical methods

A significance level of 5% was used as the alpha risk. Each estimate was given with its 95% confidence interval. Comparisons of the odds ratio and of percentages between strata were performed using their 95% confidence interval (95% CI). The primary analysis was per patient. In two studies patients were included twice, as they had FT and biopsy once before and once after the treatment; a sensitivity analysis was performed including and excluding these studies.

We used a random effects model for the primary meta-analysis to obtain a summary estimate for the AUROCs with a 95% CI of FT compared with liver biopsy.

The AUROC was used as a measure of discrimination, estimated using the empirical (non-parametric) method by DeLong et al. [18], and was compared using the paired method by Zhou et al. [19]. All analyses are performed on NCSS software (Kaysville, Utah, USA) [20].

Sensitivity analyses done by comparing AUROCs were planned for pre-specified items: study design (prospective or retrospective); analytical procedures (fresh serum or not); compliance with recommended analytical procedures (yes or no); spectrum of disease (patients with elevated or normal transaminases); year of study; co-morbidity (several morbidities including HCV, HBV, alcohol consumption, HIV coinfection, overweight, diabetes, hyperlipidemia, renal disease); whether the study was performed by the FT inventor group (yes, no, mixed groups including inventor).

Meta-analysis was performed twice, once according to the absolute value of the observed AUROCs (ObAUROC) and once according to the AUROCs standardized for the spectrum of fibrosis stages (AdAUROC). We previously demonstrated that the AUROCs were highly related to the difference between the mean fibrosis stages in the advanced fibrosis and non advanced fibrosis groups (DANA); the AdAUROC is the AUROC adjusted for the difference of the observed DANA versus a standard DANA of 2.5 fibrosis METAVIR units (DANA = 2.5 if there was a uniform prevalence of 0.20 in each of the 5 stages); all the AUROCs were adjusted to a DANA of 2.5 using the formula: AdAUROC = ObAUROC + (0.1056) (2.5-ObDANA)[11,12].

### Liver biopsies

The recruiting method of the sampling has been detailed in the previous publications. In the integrated database, liver biopsies were processed using standard techniques. A pathologist who was unaware of the biochemical markers evaluated the fibrosis stage and necrosis grade according to the METAVIR scoring system [15-17]. Fibrosis was staged on a scale of 0 – 4: F0 no fibrosis, F1 portal fibrosis without septa, F2 few septa, F3 numerous septa without cirrhosis, and F4 cirrhosis. Biopsies were performed with a 16-gauge Hepafix Luer Lock needle (Braun Melsungen) in the Paris center and the Bordeaux center, and with various needles in the multicenter study from Marseille.aucs according to the prevalence of fibrosis stages

## Results

### Databases

**Figure 1:**
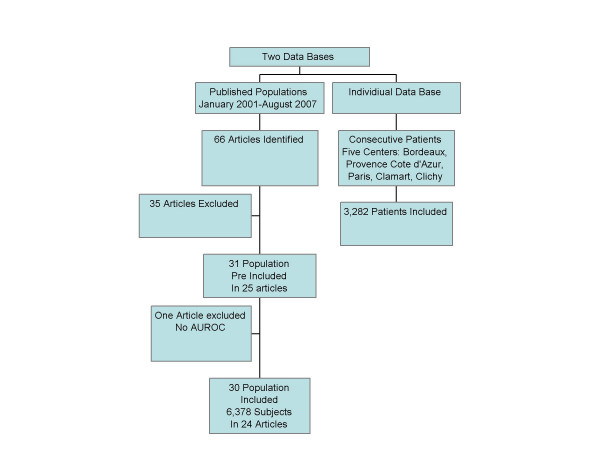
Flow sheet of the included databases.

A total of 31 studies (one population = one study) published in 25 articles between 2001 and 2007 were identified (Table 1) [3-8,21-39]; in one study, the AUROC was unknown [27] and was not included, resulting in 30 included studies (Figure 1). These included 6,378 subjects with both FT and biopsy (3,501 HCV, 1,457 HBV, 267 NAFLD, 429 ALD, and 724 mixed). One study including 208 patients with alcoholic liver disease focused on the diagnostic value of the AshTest for the diagnosis of alcoholic hepatitis [35] but the details concerning the AUROCs of FT for the diagnosis of fibrosis, previously not detailed in the original publication, were used in the present overview (Table 1).

**Table 1:** Characteristics of the FibroTest diagnostic studies for the staging of hepatic fibrosis in patients with chronic liver disease

**First author [ref]**	**Number Patients**	**Methodology**	**Age**	**Stage Prevalence**	**AUROC SE**	**F0 (%)**	**F1 (%)**	**F2 (%)**	**F3 (%)**	**F4 (%)**	**DANA**	**Size mm**	**Independent**	**Guidelines and fresh**	**Biopsy FT median days**	**High risk profile $ (%)**
**HCV n = 20**																
Imbert-1, 2001 (21)	189	Prospective Single center Training cohort	47	F2F3F4 0.38	0.84 0.03	36 (18)	91 (44)	40 (20)	18 (9)	20 (10)	2.03	16	No	Yes	0	NA
Imbert-2, 2001 (21)	134	Prospective Single center Validation cohort	48	F2F3F4 0.45	0.87 0.03	20 (15)	54 (40)	28 (21)	10 (7)	22 (16)	2.16	16	No	Yes	0	NA
Poynard-1, 2001 (22)	299	Retrospective Randomized trial Multicenter	41	F3F4 Knodell 0.36	0.74 0.03	60 (20)	133 (44)	56** (17)	55 (17)	5 (2)	1.42	NA	Mixed	Yes	NA	NA
Poynard-2, 2003 (23)	352	Retrospective Randomized trial Multicenter Before treatment	45	F2F3F4 0.39	0.73 0.03	6 (2)	206 (59)	63 (18)	32 (9)	32 (9)	1.71	17	Mixed	No	137	0 (0)
Poynard-3, 2003 (23)	352	Retrospective Randomized trial Multicenter After treatment	47	F2F3F4 0.32	0.77 0.03	15 (4)	222 (63)	64 (18)	25 (7)	26 (10)	1.73	17	Mixed	No	12	0 (0)
Rossi, 2003 (24)	125	Prospective Multicenter	40	F2F3F4 0.38	0.74 0.05	25 (20)	52 (42)	26 (21)	13 (10)	9 (7)	1.95	NA	Yes	No	0	0 (0)
Myers-1, 2003 (25)	130	Retrospective Single center HCV-HIV Co-infection	38	F2F3F4 0.45	0.86 0.04	17 (13)	55 (42)	22 (17)	19 (15)	17 (13)	2.15	NA	No	Yes	< 180	NA
Castera, 2005 (26)	183	Prospective Single center	51	F2F3F4 0.38	0.84 0.03	0 (0)	47 (26)	53 (29)	37 (20)	46 (25)	1.95	17	Yes	Yes	0	NA
Cales-2, 2005 (7)	120	Prospective Single center Validation cohort	44	F2F3F4 0.48	0.86 0.06	20 (17)	43 (36)	28 (3)	14 (12)	15 (13)	2.09	21	Yes	No	< 7	NA
Coletta, 2005 (27)	40	Prospective Multicenter PNALT	44	F2F3F4 0.35	NA	3 (8)	23 (58)	9 (23)	5 (13)	0 (0)	1.47	20	Yes	NA	180	NA
Varaut-1, 2005 (28)	50	Retrospective Single center Dialysis patients	48	F2F3F4 0.42	0.53 0.04	1 (2)	28 (56)	11 (22)	8 (16)	2 (4)	1.61	19	Yes	No	< 180	2 (4)
Varaut-2, 2005 (28)	60	Retrospective Single center Kydney recipients	44	F2F3F4 0.44	0.71 0.04	10 (21)	21 (35)	17 (28)	10 (16)	2 (3)	1.84	19	Yes	No	< 180	2 (3.3)
Halfon, 2006 (29)	504	Prospective Multicenter	45	F2F3F4 0.45	0.79 0.02	58 (12)	216 (43)	110 (22)	91 (18)	29 (6)	1.87	15	Yes	Yes	0	15 (3)
Sebastiani-1, 2006 (30)	65	Prospective PNALT	45	F2F3F4 0.39	0.71 0.04	18 (27)	22 (34)	12 (19)	6 (9)	7 (11)	2.21	18	Yes	Yes	0	Excluded
Sebastiani-2, 2006 (30)	125	Prospective EALT	50	F2F3F4 0.71	0.81 0.03	6 (5)	30 (24)	53 (42)	15 (12)	21 (17)	1.83	18	Yes	Yes	0	Excluded
Wilson, 2006 * (31)	115	Retrospective Multicenter 30% HIV	42	F2F3F4 0.38	0.74 0.05	39 (33)	36 (30)	33 (28)	8 (7)	3 (3)	1.87	11	Yes	No	Contemporaneous	NA
Sene, 2006 (32)	138	Prospective Single center Cryoglobulinemia Vasculitis	58	F2F3F4 0.47	0.83 0.03	37 (27)	36 (26)	30 (22)	16 (12)	19 (14)	3.05	18	No	Yes	< 60	11 (8)
Halfon-2, 2007 (36)	158	Prospective Single center	41	F2F3F4	0.79 0.03	6 (4)	103 (65)	34 (22)	13 (8)	2 (1)	1.38	21	Yes	Yes	0	NA
Leroy, 2007 (37)	180	Prospective Single center	44	F2F3F4	0.84 0.03	15 (8)	74 (41)	40 (22)	26 (14)	25 (14)	2.00	23	Yes	Yes	0	NA
Grigorescu, 2007 (39)	206	Single center	47	F2F3F4	0.78 0.02	16 (8)	60 (29)	70 (34)	44 (21)	16 (8)	1.80	NA	Yes	Yes	NA	Excluded
**HBV n = 4**																
Myers-2 (3)	209	Prospective and (42) Retrospective (167)	39	F2F3F4 0.29	0.78 0.04	76 (36)	72 (34)	32 (15)	10 (5)	19 (9)	2.31	18	No	Yes	1	NA
Poynard-4, 2005 (4)	214	Prospective	40	F2F3F4 0.67	0.77 0.03	14 (8)	53 (25)	119 (24)	55 (26)	40 (19)	2.17	NA	Mixed	Yes	150	7 (3.2)
Sebastiani-3, 2007 (33)	110	Prospective	43	F2F3F4	0.85 0.04	15 (14)	20 (18)	40 (36)	13 (12)	22 (20)	2.19	17	Yes	Yes	0	Excluded
Poynard-5, 2007 (34)	924	Retrospective	39	F2F3F4	0.76 0.02	21 (2)	596 (65)	160 (17)	69 (7)	78 (8)	1.77	13	Mixed	Yes	90	80 excluded (8)
**ALD n = 2**																
Naveau, 2005 (5)	221	Prospective One center	47	F2F3F4 0.64	0.84 003	16 (7)	65 (29)	48 (22)	24 (11)	68 (31)	2.34	15	Mixed	Yes	9	4 (1.8)
Thabut, 2006 (35)	208	Prospective Two centers	51	F2F3F4	0.91 0.02	10 (5)	25 (12)	21 (10)	17 (8)	136 (65)	2.98	12	Mixed	Yes	0	15 (7.2)
**NAFLD n = 2**																
Ratziu-1, 2006 (8)	170	Prospective	53	F2F3F4 0.24	0.86 0.03	77 (45)	54 (31)	20 (12)	11 (7)	9 (5)	2.30	20	No	Yes	0	5 (2.9)
Ratziu-2, 2006 (8)	97	Prospective	49	F2F3F4 0.15	0.75 0.04	26 (27)	40 (41)	15 (15)	12 (12)	4 (4)	2.04	18	Yes	Yes	0	2 (2)
**Mixed n = 3**																
Cales1, 2005 (7)	478	Prospective Single center HCV, HBV, ALD	45	F2F3F4 0.59	0.82 0.03	28 (6)	170 (36)	120 (25)	57 (12)	102 (21)	2.08	18	Yes	No	< 7	NA
Callewaert, 2004 (6)	106	Prospective HCV and ALD	NA	F4 0.29	0.89 0.04	0 (0)	28 (26)	20 (19)	10 (9)	48 (45)	2.73	NA	Yes	Yes	NA	NA
Coco, 2007 (38)	164	Prospective HCV and HBV	50	F2F3F4	0.89 0.05	29 (18)	33 (20)	13 (8)	6 (4)	83 (51)	3.15	25	Yes	Yes	90	NA

*Details for stages given out of 119 and not out of 115: ** half of Knodell F3 has been counted as METAVIR F2

NA: not available; PNALT: persistently normal ALT; EALT: Elevated ALT

$ High risk profile defined as hemolysis, Gilbert or acute inflammation; mean was 143/3,495 (4.1%)

Individual data were available in 3,282 patients who constituted the integrated data base: 2,431 HCV, 322 HBV, 267 NAFLD and 262 ALD (Table 2). Among the 3,282 patients included in the integrated database 875 patients belong to independent studies (27%), 1,431 to mixed (43%) and 976 (30%) to non-independent studies.

**Table 2:** Characteristics of the integrated data-base and FibroTest diagnostic value in liver disease

**Disease**	**Number**	**Age yr**	**Biopsy Length mm**	**F0 (%)**	**F1 (%)**	**F2 (%)**	**F3 (%)**	**F4 (%)**	**ObAUROC**	**DANA**	**AdAUROC**	**High risk profile $ (%)**
**HCV**	2,431	47	17	204 (8.4)	1123 (46.2)	531 (21.8)	298 (12.3)	275 (11.3)	0.77 0.75–0.79	1.92	0.83 0.81–0.85	26/1681 (1.5)
**HBV**	322	42	17	86 (26.7)	94 (29.2)	61 (18.9)	38 (11.8)	43 (11.4)	0.81 0.76–0.86	2.30	0.83 0.78–0.88	9/322 (2.8)
**ALD**	262	48	14	18 (6.9)	67 (25.6)	50 (19.1)	23 (8.8)	104 (39.7)	0.85 0.80–0.89*	2.52	0.85 0.80–0.89	5/262 (1.9)
**NAFLD**	267	51	19	102 (38.2)	94 (35.2)	35 (13.1)	23 (8.6)	13 (4.9)	0.81 0.74–0.86	2.21	0.84 0.77–0.89	7/267 (2.6)

**All**	3,282	47	17	410 (12.5)	1378 (42.0)	677 (20.6)	382 (11.6)	435 (13.3)	0.79 0.77–0.80	2.07	0.84 0.82–0.86	47/2,532 (1.9)

* P = 0.001 between HCV and ALD for ObAUROC, Non Significant for AdAUROC

### Comparison of FT diagnostic values between different chronic liver diseases

**Figure 2:**
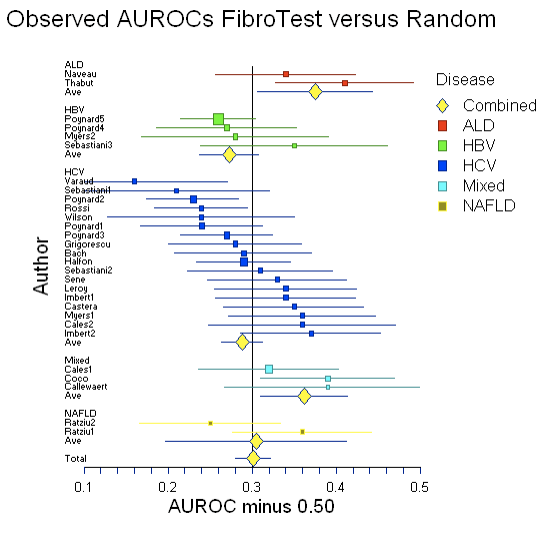
Meta-analysis of the observed area under the ROC curves (AUROC) assessed in published studies of Fibrotest diagnostic value. AUROCs were all significantly higher for Fibrotest than the random 0.50 value (upper panel) (P < 0.001). There was no significant difference between the different liver diseases.

**Figure 3:**
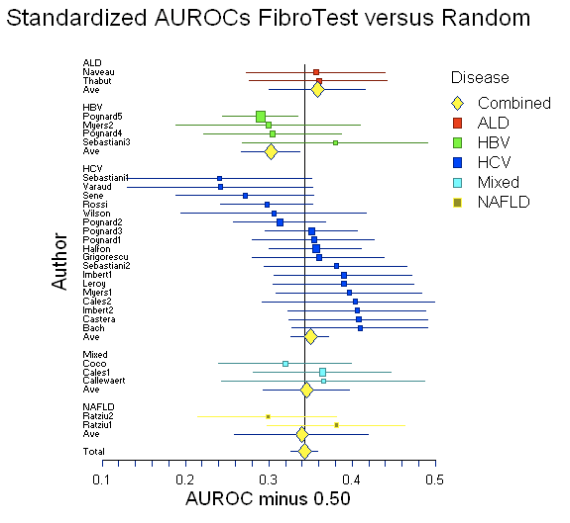
Meta-analysis of the standardized area under the ROC curves (AUROC) assessed in published studies of Fibrotest diagnostic value. There was no significant difference between the different liver diseases.

The mean of the observed AUROCs in published studies was 0.80 (95% CI, 0.78–0.82) (Figure 2) and of the AdAUROCs was 0.84 (95% CI, 0.83–0.86) (Figure 2). There was a significant heterogeneity between studies for the ObAUROCs (Cochran Q = 56; P = 0.001) but not for the AdAUROCS (Cochran Q = 26 P = 0.19). There was no significant difference between the ObAUROCs (Figure 1) or AdAUROCs (Figure 3) in HCV patients compared to other liver diseases (Table 2, and Table 3).

**Table 3:** Sensitivity analyses of FibroTest diagnostic values according to published studies

	**Observed AUROC**	**Standardized AUROCs**
Characteristic (number of studies)		
All (30)	0.80 0.78–0.82	0.84 0.83–0.86
Disease		
HCV (19)	0.79 0.76–0.82	0.85 0.82–0.87
HBV (4)	0.77 0.74–0.81	0.80 0.77–0.84
ALD (2)	0.88 0.81–0.84	0.86 0.80–0.92
NAFLD (2)	0.81 0.70–0.91	0.84 0.76–0.92
Mixed (3)	0.86 0.81–0.91	0.85 0.80–0.93
Design		
Prospective (19)	0.83 0.81–0.85*	0.86 0.84–0.88
Retrospective (11)	0.76 0.73–0.78	0.82 0.80–0.84
Authors		
Independent (16)	0.80 0.77–0.83	0.85 0.82–0.88
Mixed (5)	0.76 0.73–0.80	0.83 0.81–0.86
Inventor (9)	0.83 0.79–0.87	0.84 0.81–0.88
Guidelines/Fresh		
No (7)	0.77 0.72–0.79	0.83 0.80–0.86
Yes (23)	0.82 0.79–0.84**	0.84 0.83–0.86
Length biopsy		
< 18 (6)	0.81 0.76–0.86	0.84 0.80–0.87
> = 18 (18)	0.81 0.78–0.84	0.85 0.83–0.87
Interval serum-biopsy		
> 30 days (8)	0.79 0.74–0.84	0.81 0.78–0.84
< 30 (15)	0.80 0.78–0.83	0.85 0.83–0.87
Co-morbidity		
No (21)	0.79 0.77–0.81	0.85 0.83–0.87
Yes (8)	0.83 0.78–0.88	0.83 0.80–0.87

* P = 0.001 ** P = 0.05

In the integrated data base, the mean FT ObAUROC was 0.79 (95% CI, 0.77–0.82) and the mean AdAUROC was 0.84 (0.82–0.86). There was no significant difference between AdAUROCs in HCV patients compared to other liver diseases. The only significant difference was a higher ObAUROC in ALD than in HCV (P = 0.001) (Table 2).

Sensitivity analyses according to study characteristics are detailed in Table 3 for meta-analysis and in Table 4 for the integrated data base. There were no significant differences according to liver disease, baseline transaminases level, authors' independency, to the mean length of biopsy, the interval serum-biopsy, and co-morbidity. Prospective studies, and studies following guidelines were associated with higher ObAUROCs but these differences were no more significant for AdAUROCs. In the integrated database fragmented biopsies were associated with higher ObAUROC but this difference was no more significant for AdAUROCs.

**Table 4:** Sensitivity analyses of FibroTest diagnostic values according to individual data

**Characteristic (number of patients)**	**Observed AUROC**	**DANA**	**Adjusted AUROC**
Length biopsy			
< 25 mm (2,446)	0.80 0.78–0.82	2.10	0.84 0.82–0.86
> = 25 mm (492)	0.77 0.72–0.81	1.91	0.83 0.78–0.87
More than 2 fragments			
Yes (575)	0.86 0.82–0.89*	2.45	0.87 0.83–0.90
No (606)	0.78 0.73–0.81	2.05	0.83 0.78–0.86
Normal baseline ALT			
No (1,833)	0.80 0.78–0.82	2.16	0.84 0.82–0.86
Yes (493)	0.79 0.74–0.84	2.39	0.81 0.76–0.86

*P = 0.002

### Comparison of FT diagnostic values between adjacent stages

The AUROC for the diagnosis of intermediate stages F2 vs. F1 (0.66; 0.63–0.68, n = 2,055) was not different compared to the extreme stages: F3 vs. F4 (0.69; 0.65–0.72, n = 817) or F1 vs. F0 (0.62; 0.59–0.65, n = 1788). There were also no differences between adjacent stages when the AUROCs were compared for each chronic liver disease (Table 5).

**Table 5:** FibroTest diagnostic values between adjacent stages

	**Observed AUROC**
Adjacent stages	
F1 vs. F0 (1,788)	0.62 0.59–0.65
HCV (1,327)	0.64 0.60–0.68
HBV (180)	0.64 0.56–0.72
ALD (85)	0.47 0.32–0.60
NAFLD (196)	0.53 0.45–0.61
F2 vs. F1(n = 2,055)	0.66 0.63–0.68
HCV (1,654)	0.66 0.63–0.69
HBV (155)	0.63 0.53–0.71
ALD (117)	0.65 0.53–0.74
NAFLD (129)	0.69 0.57–0.78
F3 vs. F2 (n = 1,059)	0.67 0.64–0.70
HCV (829)	0.66 0.62–0.69
HBV (99)	0.78 0.67–0.86
ALD (73)	0.66 0.50–0.77
NAFLD (58)	0.69 0.52–0.80
F4 vs F3 (817)	0.69 0.65–0.72
HCV (573)	0.66 0.61–0.70
HBV (81)*	0.54 0.40–0.65
ALD (127)*	0.82 0.69–0.90
NAFLD (36)	0.71 0.45–0.86

* P = 0.001 between HBV and ALD

### High risk profile

The overall prevalence of patients with high risk profile of false positive or false negative (suspected Gilbert syndrome, hemolysis and acute inflammation) was 4.1% (143/3,495; 95%CI 3.5%–4.8%) among the studies and 1.9% in the integrated data-base (47/2,532 95%CI 1.4%–2.5%).

## Discussion

This meta-analysis demonstrated that the diagnostic value of FT was similar in the four most frequent chronic liver diseases. This meta-analysis also demonstrated that the diagnostic value of FT, as for liver biopsy, was similar between all the adjacent fibrosis stages but without a specific "gray zone" or "inaccurate zone" between intermediate stages. FT, like biopsy, has lower diagnostic value to discriminate between two adjacent stages than between two extreme stages [17].

The advantages of the present study are the large number of studies included, as well as the opportunity to analyze an integrated database, which included the individual characteristics of 3,282 (51%) patients out of 6,378 patients included in the published studies. This permitted to better take into-account the variability factors associated with FT diagnostic value.

### Comparison of FT diagnostic values between different chronic liver diseases

One limitation of the study is that the number of studies and patients in non HCV related diseases is smaller than those in HCV. However we analyzed a total of eleven studies including 2,877 non HCV or mixed causes, and 851 non-HCV patients in the integrated data base. Another limitation was that there were few independent studies in other chronic liver diseases (1 for HBV, 1 for NAFLD and none for ALD). However two studies in HBV [4,34] and two studies in ALD [5,35] were mixed and three independent studies included HBV and ALD in their analyses [6,7,38] with same results than in non-independent studies (Table 1).

To compare as well as possible the FT diagnostic value according to liver diseases, we used the standardization of the AUROCs, and sensitivity analysis in both the meta-analysis and the integrated data base with individual data.

We applied the standardization of the observed AUROCs according to the spectrum of fibrosis stages among advanced and non advanced fibrosis. We recently demonstrated that this standardization is mandatory for any interpretation of AUROCs estimating the diagnostic value of a fibrosis marker [12]. For instance, this method allowed an adjustment to be made in the ObAUROCs of FT according to the cause of liver disease, which had significant difference in fibrosis stage spectrum. The significant difference observed between ALD and HCV ObAUROCs disappeared after adjustment (Table 2). In HBV studies patients had lower DANA than in studies of ALD patients. After standardization, the difference between AUROCs was reduced by two (0.77 versus 0.88 before and 0.80 versus 0.86 after standardization) (Table 2).

These data are also in accordance with the similarities of advanced fibrosis stages among chronic hepatitis C and B, NAFLD and ALD. Despite differences in the dynamics of fibrosis progression [40] and the initial fibrosis stages, the bridging stages are very similar including cirrhosis and were estimated in the same way by the METAVIR scoring system for advanced fibrosis [40,41]. Fibrosis stages and pathogenetic mechanisms are very similar in NAFLD and ALD [42]. Repeated FT improved similar to fibrosis as estimated by repeated biopsies during treatment for HCV [22,23], HBV [4,34] and NAFLD [43]. The components of the FT had similar modifications according to fibrosis stages for these four chronic liver diseases [1,3,5,8].

The sensitivity analyses did not reveal any significant differences between AdAUROCs according to all the other characteristics analyzed (Table 3 and Table 4). Significant differences or the absence of differences between ObAUROCs could be due to confounding factors. A demonstrative illustration is the artificially higher ObAUROCs for fragmented versus non-fragmented biopsies. Because of a higher prevalence of cirrhosis in patients with fragmented biopsies, the DANA was higher than in patients with non-fragmented biopsies [11]. This difference was no longer seen after adjustment [Table 4).

### Comparison of FT diagnostic values between adjacent stages

There is still a controversy among experts concerning the FT diagnostic value for "intermediate fibrosis stages". For panel biomarkers including FT, Gebo et al. stated that "One of the major limitations may be in the lack of reliable identification and classification of the intermediate stages of fibrosis"[9]. Bissell also stated that for panels including FT "Their accuracy for intermediate fibrosis is relatively poor." [44]. Rockey and Bissell stated that "Decision-making requires a test that differentiates minimal disease [stage 0/1 fibrosis) from intermediate fibrosis [stage 2/3). For this purpose, the current generation of non-invasive tests falls short, and liver biopsy still is needed for definitive staging" [45]. These statements are not evidence-based.

The first error is stating that "liver biopsy is still needed for definitive staging of intermediate stages". The entire liver is certainly the gold standard but the liver biopsy is an imperfect gold standard. The present overview of the 25 studies giving biopsy length, all performed in tertiary centers, observed among 5,404 patients that the median of mean biopsy length was 18 mm. For the two larger studies including more than 500 patients (total 1,428) the median was 14 mm and in the integrated data base the mean was 17 mm out of 3,282. In our tertiary center a prospective study observed in 1,769 patients that biopsy was greater than 25 mm in only 16% (280/1769) of patients [46].

A liver biopsy of 15 mm has an AUROC of 0.82 between F1 and F2, being around 20% of false positives or false negatives [17]. Therefore FT with an AUROC of 0.66 (usually described as a weak value when using a true gold standard) between F1 and F2 has a relative AUROC versus the best AUROC possible of 0.66/0.82 = 0.80, which is in the end acceptable for a non invasive test.

The second error is the confusion between intermediate stages and adjacent stages. For any estimate of liver fibrosis, the diagnostic values (AUROCs) between adjacent stages need to be assessed. There are no significant differences in the diagnostic values (AUROCs) for FT as demonstrated in this study, or for liver biopsy as demonstrated by Bedossa et al [17] according to intermediate stages as opposed to extreme stages, with the AUROCs for all adjacent stages being similar. The same results have been observed for all the combinations of stages in a previous study [1] and with this database (data not showed).

The third error is assessing the diagnostic value of a biomarker in a subpopulation of patients defined by liver biopsy such as F2/F3 vs. F0/F1. The exclusion of F4 patients defined by a 15 mm biopsy will not exclude the risk of false positives or false negatives of the remaining non-F4. It is much more important to assess the spectrum of fibrosis stage among the F0/F1 and F2/F3; if the AUROCs are not standardized according to the DANA, the ObAUROCs will be misleading [12]. This once again underlines that assessing the AUROCs between all adjacent stages remains the best way, knowing that for the "perfect" biomarker, the best possible achievable AUROC is 0.82 for a 15 mm biopsy.

There are also different methodological approaches for the overview of fibrosis markers. Parkes et al. arbitrarily defined an "inaccurate" zone of a marker when it "cannot reliably attribute result for tests as tests perform with lower sensitivities/specificities at thresholds, where positive predictive value < 90%, negative predictive value > 95%" [47]. There is no rationale for choosing these thresholds, but this definition could be acceptable if a true gold standard existed. This is not the case for fibrosis markers. If this definition is applied to 15 mm liver biopsies, the biopsy will be inaccurate in 40% of cases for a diagnosis between F1 and F2.

The only significant difference identified using AUROCs between adjacent stages (Table 5) was for HBV versus ALD. The obAUROC for ALD was particularly high and this should be validated in population with greater sample size.

### High risk profile

"The observed high risk profile of FT in the published studies (4.1%) and in the integrated database (1.9%) were concordant with the post marketing analyses finding (2.1%) in 32,527 consecutive tests [2,46]. In these analyzes there were 272 cases (0.8%) with a high-risk profile of false positives, for which the other components were not concordant in favor of significant fibrosis. Patients with extremely low haptoglobin, particularly when the rest of the exams were hardly modified, could have had hemolysis. A high-risk profile of false positives due to possible Gilbert syndrome was observed in 409 (1.3%) cases. In the presence of acute inflammation (i.e., sepsis or acute hemolysis), FT analysis must be postponed [2]."

## Conclusion

This study suggests that FT could be used as an alternative to liver biopsy in the four more common chronic liver diseases: HCV, HBV, NAFLD and ALD. Neither biomarkers nor biopsy are sufficient alone to take definitive decision in a given patient and all the clinical and biological data must be taken into account.

However, due to the dramatically insufficient risk-benefit ratio of biopsy (coefficient variation 40%, 0.3% severe adverse events and 3/10,000 mortality), it is surprising that many leaders and associations in the field of hepatology still recommend liver biopsy as the first line investigation for millions of people exposed to the risk of fibrosis. This study reinforced our previous conclusion [48] that, based on current evidence, a wise recommendation would be a moratorium on liver biopsy as a first line procedure while awaiting studies demonstrating its cost-utility versus that of biomarkers. Biopsy as a second line estimate of liver injury should still be indicated for intricate diseases or clinicobiological discordances.

Practices are evolving rapidly and in France a nationwide survey recently found that among 546 hepatologists, 81% used non-invasive biomarker (FibroTest-ActiTest) and 32% used elastography, with a dramatic decrease in the use of liver biopsy for more than 50% of patients with chronic hepatitis C, and with a subsequent increase in the number of patients treated [49]. FibroTest is available in more than 50 countries [50] and the cost varies (from 100 to 300 euros per country) according to the price of the five components and the price of algorithms. In France the cost of the components was covered by social security since 2002 and the algorithms reimbursement has been approved in December 2006 [51].

A recent overview by French health authorities officially approved non invasive biomarkers FibroTest and elastography (Fibroscan) as first line estimates of fibrosis in patients with chronic hepatitis C, recommended reimbursement by social security and approved liver biopsy only as second line estimate in case of discordance or non interpretability of non invasive markers. An updated overview is pending for other chronic liver diseases at the end of 2007 [50].

## Competing interests

TP is a consultant and has a capital interest in Biopredictive, the company marketing FT, and MM is a full time employee of Biopredictive.

Biopredictive had no role in the study design, data collection, data analysis, data interpretation, or writing of the report. The corresponding author had full access to all the data in the study and had final responsibility for the decision to submit for publication.

## Authors' contributions

TP conceived the study, performed the statistical analysis, and wrote the manuscript. RM, PH, LC, VR, FIM, SN, DT, DL, FZ, MB, PC, MM, and VdL participated in the coordination of the study, data monitoring and drafted the manuscript. All authors read and approved the final manuscript.

## Pre-publication history

The pre-publication history for this paper can be accessed here:



## References

[B1] Poynard T, Imbert-Bismut F, Munteanu M, Messous D, Myers RP, Thabut D, Ratziu V, Mercadier A, Benhamou Y, Hainque B (2004). Overview of the diagnostic value of biochemical markers of liver fibrosis (FibroTest, HCV-Fibrosure) and necrosis (ActiTest) in patients with chronic hepatitis C. Comp Hepatol.

[B2] Poynard T, Imbert-Bismut F, Munteanu M, Ratziu V (2005). FibroTest-FibroSURE:towards a universal biomarker of liver fibrosis?. Expert Rev Mol Diag.

[B3] Myers RP, Tainturier MH, Ratziu V, Piton A, Thibault V, Imbert-Bismut F, Messous D, Charlotte F, Di Martino V, Benhamou Y, Poynard T (2003). Prediction of liver histological lesions with biochemical markers in patients with chronic hepatitis B. J Hepatol.

[B4] Poynard T, Zoulim F, Ratziu V, Degos F, Imbert-Bismut F, Deny P, Landais P, El Hasnaoui A, Slama A, Blin P, Thibault V, Parvaz P, Munteanu M, Trepo C (2005). Longitudinal assessment of histology surrogate markers (Fibrotest-Actitest) during lamivudine therapy in patients with chronic hepatitis B infection. Am J Gast.

[B5] Naveau S, Raynard B, Ratziu V, Abella A, Imbert-Bismut F, Messous D, Beuzen F, Capron F, Thabut D, Munteanu M, Chaput JC, Poynard T (2005). Biomarkers for the prediction of liver fibrosis in patients with chronic alcoholic liver disease. Clin Gastroenterol Hepatol.

[B6] Callewaert N, Van Vlierberghe H, Van Hecke A, Laroy W, Delanghe J, Contreras R (2004). Noninvasive diagnosis of liver cirrhosis using DNA sequencer-based total serum protein glycomics. Nature Med.

[B7] Cales P, Oberti F, Michalak S, Hubert-Fouchard I, Rousselet MC, Konate A, Gallois Y, Ternisien C, Chevailler A, Lunel F (2005). A novel panel of blood markers to assess the degree of liver fibrosis. Hepatology.

[B8] Ratziu V, Massard J, Charlotte F, Messous D, Imbert-Bismut F, Bonyhay L, Tahiri M, Munteanu M, Thabut D, Cadranel JF, Le Bail B, De Ledinghen V, Poynard T, the LIDO Study Group and the CYTOL Study Group (2006). Diagnostic value of biochemical markers (FibroTest-FibroSURE) for the prediction of liver fibrosis in patients with non-alcoholic fatty liver disease. BMC Gastroenterology.

[B9] Gebo KA, Herlong HF, Torbenson MS, Jenckes MW, Chander G, Ghanem KG, El-Kamary SS, Sulkowski M, Bass EB (2002). Role of liver biopsy in management of chronic hepatitis C: A systematic review. Hepatology.

[B10] Sebastiani G, Alberti A (2006). Non invasive fibrosis biomarkers reduce but not substitute the need for liver biopsy. World J Gastroenterol.

[B11] Poynard T, Halfon P, Castera L, Charlotte F, Bail BL, Munteanu M, Messous D, Ratziu V, Benhamou Y, Bourliere M, Ledinghen VD, the Fibropaca group (2007). Variability of the area under the receiver operating characteristic curves in the diagnostic evaluation of liver fibrosis markers: impact of biopsy length and fragmentation. Aliment Pharmacol Ther.

[B12] Poynard T, Halfon P, Castera L, Munteanu M, Imbert-Bismut F, Ratziu V, Benhamou Y, Bourliere M, de Ledinghen V, FibroPaca Group (2007). Standardization of ROC curve areas for diagnostic fvaluation of liver fibrosis markers based on prevalences of fibrosis Stages. Clin Chem.

[B13] Bossuyt PM, Reitsma JB, Bruns DE (2003). Towards complete and accurate reporting of studies of diagnostic accuracy: the STARD initiative. Clin Radiol.

[B14] Easterbrook PJ, Berlin JA, Gopalan R, Matthews DR (1991). Publication bias in clinical research. Lancet.

[B15] The French METAVIR Cooperative Study Group (1994). Intraobserver and interobserver variations in liver biopsy interpretation in patients with chronic hepatitis C. Hepatology.

[B16] Bedossa P, Poynard T (1996). An algorithm for the grading of activity in chronic hepatitis C. The METAVIR Cooperative Study Group. Hepatology.

[B17] Bedossa P, Dargère D, Paradis V (2003). Sampling variability of liver fibrosis in chronic hepatitis C. Hepatology.

[B18] DeLong ER, DeLong DM, Clarke-Pearson DL (1988). Comparing the areas under two or more correlated receiver operating characteristic curves: a nonparametric approach. Biometrics.

[B19] Zhou X, Obuchowski N, McClish D (2002). Statistical Methods in Diagnostic Medicine.

[B20] Hintze JL (2007). NCSS 2007 User Guide. Number Cruncher Statistical Systems software. NCSS, Kaysville, Utah.

[B21] Imbert-Bismut F, Ratziu V, Laurence Pieroni L, Charlotte F, Benhamou Y, Poynard T, MULTIVIRC Group (2001). Biochemical markers of liver fibrosis in patients with hepatitis C virus infection: a prospective study. Lancet.

[B22] Poynard T, Imbert-Bismut F, Ratziu V, Chevret S, Jardel C, Moussalli J, Messous D, Degos F (2002). Biochemical markers of liver fibrosis in patients infected by Hepatitis C Virus: Longitudinal validation in a randomized trial. J Viral Hepatitis.

[B23] Poynard T, McHutchison J, Manns M, Myers RP, Albrecht J (2003). Biochemical surrogate markers of liver fibrosis and activity in a randomized trial of peginterferon alfa-2b and ribavirin. Hepatology.

[B24] Rossi E, Adams L, Prins A, Bulsara M, de Boer B, Garas G, MacQuillan G, Speers D, Jeffrey G (2003). Validation of the FibroTest biochemical markers score in assessing liver fibrosis in hepatitis C patients. Clin Chem.

[B25] Myers RP, Benhamou Y, Imbert-Bismut F, Thibault V, Bochet M, Charlotte F, Ratziu V, Bricaire F, Katlama C, Poynard T (2003). Serum biochemical markers accurately predict liver fibrosis in HIV and hepatitis C virus-coinfected patients. AIDS.

[B26] Castéra L, Vergniol J, Foucher J, Brigitte Le Bail B, Chanteloup E, Haaser M, Darriet M, Couzigou P, de Lédinghen V (2005). Prospective comparison of transient elastography, Fibrotest, APRI and liver biopsy for the assessment of fibrosis in chronic hepatitis C. Gastroenterology.

[B27] Colletta C, Smirne C, Fabris C, Toniutto P, Rapetti R, Minisini R, Pirisi M (2005). Value of two noninvasive methods to detect progression of fibrosis among HCV carriers with normal aminotransferases. Hepatology.

[B28] Varaut A, Fontaine H, Serpaggi J, Verkarre V, Vallet-Pichard A, Nalpas B, Imbert Bismuth F, Lebray P, Pol S (2005). Diagnostic accuracy of the fibrotest in hemodialysis and renal transplant patients with chronic hepatitis C virus. Transplantation.

[B29] Halfon P, Bourliere M, Deydier R, Portal I, Renou R, Bertrand J, Trana A, Rosenthal A, Rotily M, Sattonet A (2006). Independent prospective multicenter validation of biochemical markers (Fibrotest-Actitest) for the prediction of liver fibrosis and activity in patients with chronic hepatitis C. Am J Gastro.

[B30] Sebastiani G, Vario A, Guido M, Noventa F, Plebani M, Pistis R, Ferrari A, Alberti A (2006). Stepwise combination algorithms of non-invasive markers to diagnose significant fibrosis in chronic hepatitis C. J Hepatol.

[B31] Wilson LE, Torbenson M, Astemborski J, Faruki H, Spoler C, Rai R, Mehta S, Kirk GD, Nelson K, Afdhal N, Thomas DL (2006). Progression of liver fibrosis among injection drug users with chronic hepatitis C. Hepatology.

[B32] Sène D, Limal N, Djamila Messous D, Ghillani-Dalbin P, Charlotte F, Halfon P, Jean-Marie Thiollière JM, Piette JC, Imbert-Bismut F, Halfon P, Poynard T, Cacoub P (2006). Biological markers of liver fibrosis and activity as non-invasive alternatives to liver biopsy in patients with chronic hepatitis C and associated mixed cryoglobulinemia vasculitis. Clin Biochem.

[B33] Sebastiani G, Vario A, Guido M, Alberti A (2007). Sequential algorithms combining non-invasive markers and biopsy for the assessment of liver fibrosis in chronic hepatitis B. World J Gastroenterol.

[B34] Poynard T, Ngo Y, Marcellin P, Hadziyannis S, Goodman Z, Ratziu V, Benhamou Y, Brosgart CL, Adefovir Dipivoxil 437 and 438 Study Groups (2007). Impact of adefovir dipivoxil on liver fibrosis and activity assessed with FibroTest-ActiTest in patients with chronic hepatitis B infection. Abstract EASL, J Hepatol.

[B35] Thabut D, Naveau S, Charlotte F, Massard J, Ratziu V, Imbert-Bismut F, Cazals-Hatem D, Abella A, Messous D, Beuzen F, Munteanu M, Taieb J, Moreau R, Lebrec D, Poynard T (2006). The diagnostic value of biomarkers (AshTest) for the prediction of alcoholic steato-hepatitis in patients with chronic alcoholic liver disease. J Hepatol.

[B36] Halfon P, Bacq Y, De Muret A, Penaranda G, Bourliere M, Ouzan D, Tran A, Botta D, Renou C, Brechot MC, Degott C, Paradis V (2007). Comparison of test performance profile for blood tests of liver fibrosis in chronic hepatitis C. J Hepatol.

[B37] Leroy V, Hilleret MN, Sturm N, Trocme C, Renversez JC, Faure P, Morel F, Zarski JP (2007). Prospective comparison of six non-invasive scores for the diagnosis of liver fibrosis in chronic hepatitis C. J Hepatol.

[B38] Coco B, Oliveri F, Maina AM, Ciccorossi P, Sacco R, Colombatto P, Bonino F, Brunetto MR (2007). Transient elastography: a new surrogate marker of liver fibrosis influenced by major changes of transaminases. J Vir Hep.

[B39] Grigorescu M, Rusu M, Neculoiu D, Radu C, Aerban A, Caþanao M, Grigorescu MD (2007). The FibroTest Value in Discriminating between Insignificant and Significant Fibrosis in Chronic Hepatitis C Patients. The Romanian Experience. Gastrointestin Liver Dis.

[B40] Poynard T, Mathurin P, Lai CL, Guyader D, Poupon R, Tainturier MH, Myers RP, Muntenau M, Ratziu V, Manns M, Vogel A, Capron F, Chedid A, Bedossa P, PANFIBROSIS Group (2003). A comparison of fibrosis progression in chronic liver diseases. J Hepatol.

[B41] Michalak S, Rousselet MC, Bedossa P, Pilette C, Chappard D, Oberti F, Gallois Y, Cales P (2003). Respective roles of porto-septal fibrosis and centrilobular fibrosis in alcoholic liver disease. J Pathol.

[B42] Lieber CS (2004). CYP2E1: from ASH to NASH. Hepatol Res.

[B43] Munteanu M, Poynard T, Charlotte F, Jacqueminet S, Messous D, Podevin P, Serfaty L, Bruckert E, Grimaldi A, Ratziu V (2007). Utility of a combination of non-invasive biomarkers (Fibromax) in assessing the efficacy of rosiglitazone in a one year randomized, double-blind trial in non alcoholic steatohepatitis. Abstract EASL, J Hepatol.

[B44] Bissell MD (2004). Assessing fibrosis without a liver biopsy: are we there yet?. Gastroenterology.

[B45] Rockey DC, Bissell MD (2006). Noninvasive measures of liver fibrosis. Hepatology.

[B46] Poynard T, Munteanu M, Imbert-Bismut F, Charlotte F, Thabut D, Le Calvez S, Messous D, Thibault V, Benhamou Y, Moussalli J, Ratziu V (2004). Prospective Analysis of Discordant Results between Biochemical Markers and Biopsy in Patients with Chronic Hepatitis C. Clin Chem.

[B47] Parkes J, Indra Neil Guha IN, Roderick P, Rosenberg W (2006). Performance of serum marker panels for liver fibrosis in chronic hepatitis C. J Hepatol.

[B48] Poynard T, Ratziu V, Benhamou Y, Thabut D, Moussalli J (2005). Biomarkers as a first-line estimate of injury in chronic liver diseases: time for a moratorium on liver biopsy?. Gastroenterology.

[B49] Castera L, Denis J, Babany G, Roudot-Thoraval F (2007). Evolving practices of non-invasive markers of liver fibrosis in patients with chronic hepatitis C in France: Time for new guidelines?. J Hepatol.

[B50] http://www.biopredictive.com.

[B51] La Haute Autorité de Santé (HAS) in France The HAS recommendations for the managemeMonant of the chronic hepatitis C using non-invasive biomarkers. http://www.has-sante.fr/portail/display.jsp?id=c_476486.

